# Early Toll-like receptor 4 inhibition improves immune dysfunction in the hippocampus after hypoxic-ischemic brain damage

**DOI:** 10.7150/ijms.66494

**Published:** 2022-01-01

**Authors:** Zhu Xing, Tang Zhen, Fan Jie, Yu Jie, Liu Shiqi, Zhu Kaiyi, OuYang Zhicui, Hei Mingyan

**Affiliations:** 1 Department of Neonatology, Neonatal Center, Beijing Children's Hospital, Capital Medical University, Beijing, 100045 China.; 2 Department of Neonatology, Affiliated Hospital of Guilin Medical College, Guilin, Guangxi, 541001 China.; 3 Department of Pediatrics, the Third Xiangya Hospital of Central South University, Changsha, Hunan, 410013 China.; 4 Department of Neonatology, East Hospital of Shaoyang Central Hospital, Shaoyang, Hunan, 422000 China.

**Keywords:** Hypoxic-ischemic, Brain damage, Hippocampus, Toll-like receptor 4, Rats, Neuroimmune

## Abstract

**Background**: Toll-like receptor 4 (TLR4) is implicated in neonatal hypoxic-ischemic brain damage (HIBD), but the underlying mechanism is unclear.

**Hypothesis**: We hypothesized that TLR4 mediates brain damage after hypoxic ischemia (HI) by inducing abnormal neuroimmune responses, including activation of immune cells and expression disorder of immune factors, while early inhibition of TLR4 can alleviate the neuroimmune dysfunction.

**Method**: Postnatal day 7 rats were randomized into control, HI, and HI+TAK-242 (TAK-242) groups. The HIBD model was developed using the Rice-Vannucci method (the left side was the ipsilateral side of HI). TAK-242 (0.5 mg/kg) was given to rat pups in the TAK-242 group at 30 min before modeling. Immunofluorescence, immunohistochemistry, and western blotting were used to determine the TLR4 expression; the number of Iba-1+, GFAP+, CD161+, MPO+, and CD3+ cells; ICAM-1 and C3a expression; and interleukin (IL)-1β, tumor necrosis factor (TNF)-α, and IL-10 expression in the hippocampal CA1 region.

**Result:** Significantly increased TLR4 expression was observed in the left hippocampus, and was alleviated by TAK-242. The significant increases in Iba-1+, MPO+, and CD161+ cells at 24 h and 7 days after HI and in GFAP+ and CD3+ T cells at 7 days after HI were also counteracted by TAK-242, but no significant differences were observed among groups at 24 h after HI. ICAM-1 expression increased 24 h after HI, while C3a expression decreased; TAK-242 also alleviated these changes. TNF-α and IL-1β expression increased, while IL-10 expression decreased at 24 h and 7 days after HI; TAK-242 counteracted the increased TNF-α and IL-1β expression at 24 h and the changes in IL-1β and IL-10 at 7 days, but induced no significant differences in IL-10 expression at 24 h and TNF-α expression at 7 days.

**Conclusion:** Early TLR4 inhibition can alleviate hippocampal immune dysfunction after neonatal HIBD.

## Introduction

The central nervous system (CNS) has been regarded as an immune-privileged site, however, mounting evidence has shown that this privilege is relative. Neuroimmunity plays an important role in brain development, damage and repair 1-4. Neonatal hypoxic-ischemic brain damage (HIBD), mainly caused by perinatal asphyxia, is a severe neurological damage disease in term neonates, involving multiple underlying mechanisms and pathways 5, 6. The role of neuroimmune dysfunction in HIBD has been emphasized. The activation of various immune cells and the abnormal immune factors are important in promoting the acute cell death and long-term neurological damage in HIBD. However, the underlying mechanisms are unclear.

Toll-like receptor 4 (TLR4) is widely distributed in CNS, expressed in astrocytes, microglia, vascular smooth muscle cells and endothelial cells, and it is an important pattern recognition receptor (PRR) on nerve cell membranes 7.On exposure to conditions such as ischemia, TLR4 expression is upregulated, which amplifies the neuroinflammation cascade via multiple signaling pathways 8-13. TLR4 inhibition elicits neuroprotective effects against brain damage 12, 14, 15. Our previous studies found that the expression of TLR4 in the hippocampus increased at 24 h after HI, and the early suppression of TLR4 expression mitigated the loss of neurons and improved the behavior of adolescent rats 16. However, the mechanism underlying neurological damage caused by TLR4 through the neuroimmune has not been established.

We hypothesized that TLR4 mediates brain damage after HI by inducing abnormal neuroimmune responses in the CNS, including activation of resident and peripheral immune cells and expression disorder of inflammatory factors, adhesion factors, and the complement system, while early inhibition of TLR4 can alleviate the neuroimmune dysfunction. Thus, we investigated the changes of various immune cells and immune factors in the hippocampi of neonatal HIBD model rats after early TLR4 inhibition to determine the mechanism underlying TLR4-mediated damage after HI.

## Materials and Methods

### Animals and HIBD Modeling

Postnatal day 7 (P7) pups (weight: 13-19 g) were housed under standard conditions (25.0 ± 1.0 °C, 12/12 h light/dark cycle) in specific pathogen-free animal quarters with food and water *ad libitum*. Every effort was made to minimize animal suffering and reduce the number of animals used in our experiments.

The HIBD model was established according to the Rice-Vannucci method 17. The animals were anaesthetized using isoflurane (4% induction, 2% maintenance) carried by O_2_, and the left common carotid artery was exposed and dissected after double ligation with a 5-0 suture. The duration of surgery was approximately 5 min for each pup. After recovery for 1 h, the pups were exposed to a hypoxic chamber with 8% O_2_ and 92% N_2_ for 2 h at 37 °C. The pups were returned to the dam once hypoxia exposure was completed. The pups in the control group received anesthesia and were subjected to common carotid artery exposure with neither ligation nor hypoxia.

### Experimental Design and TAK-242 Administration

Rat pups without gender difference (*χ^2^* = 2.027, *P* = 0.363) were randomized into three groups: control, HI, and HI + TAK-242 (TAK-242) (Table 1). TAK-242 (MedChemExpress, USA) was dissolved in 1% dimethyl sulfoxide (DMSO) with a final concentration of 0.1 mg/mL. The TAK-242 group received an intraperitoneal injection of TAK-242 (0.5 mg/kg) at 30 min before HI. The control and HI groups received intraperitoneal injections of saline containing the same volume and concentration of DMSO.

### Tissue Preparation

Rat pups were euthanized at 6 h, 24 h, and 7 days after HI. Animals for morphological observation were perfused transcardially with phosphate buffered saline (PBS) and 4% paraformaldehyde sequentially. The brains were removed, post-fixed in 4% paraformaldehyde, paraffin-embedded, and sliced (Leica CM1510S, Germany). For western blotting, the ipsilateral (left) hippocampal tissues were isolated and pooled for each group. Subsequently, the samples were lysed using a mixture of radioimmunoprecipitation assay lysis buffer (Beyotime Biotechnology, China) and proteinase inhibitor (99:1; Sigma-Aldrich). After centrifugation at 2000 rpm for 10 min at 4 °C, the supernatant was collected and stored at -80 °C until further use.

### Immunohistochemistry and Immunofluorescence

The 5 μm sections for immunohistochemistry were immersed in xylene and alcohol gradient and boiled for 20 min in citric acid (pH 6.0). After natural cooling, the sections were immersed in 3% H_2_O_2_ for 10 min, and in 5% goat serum for 30 min at 37 ℃ in sequence. Subsequently, the sections were incubated with a primary antibody overnight at 4°C, followed by incubation with goat anti-mouse IgG H&L (HRP) for 30 min at 37 °C. Finally, a color reaction was performed using a diaminobenzidine chromogen kit (Zhongshan Goldenbridge Biotechnology, China) and counterstaining was performed using hematoxylin. Three fields were randomly selected from the hippocampal CA1 region of each animal under a light microscope, and Image-Pro Plus 6.0 was used to analyze the percentage of the positive area ([IOD/area] × 100%). The primary antibodies included anti-TLR4 (1:200, Abcam), anti-ICAM-1 (1:100, Abcam), and anti-C3a (1:100, ABclonal).

The sections for immunofluorescence were directly blocked with 5% goat serum for 30 min after antigen repair and subsequently incubated with primary antibodies overnight at 4 °C. After washing with PBS, the sections were incubated with the corresponding Alexa Fluor® 594 conjugated antibodies (1:200, Jackson ImmunoResearch Laboratories, Inc.) for 1 h. Finally, the sections were covered with DAPI and sealed with an anti-quenching agent. The slides were visualized with a fluorescence microscope (Olympus FluoviewTM FV1000, Olympus Corporation, Japan) and analyzed using Image-Pro Plus 6.0. Three fields were randomly selected from the hippocampal CA1 region of each animal and the number of positive cells per field was evaluated. The primary antibodies included anti-Iba-1 (1:200, Abcam), anti-GFAP (1:200, Abcam), anti-CD161(1:200, Serotec), anti-MPO (1:200, Abcam), anti-CD3(1:200, Abcam).

### Western Blotting

The protein concentrations were determined using an enhanced BCA protein assay kit (Proteintech, China). Samples containing equivalent amounts of protein (30 μg) were subjected to 8-12% sodium dodecyl sulfate-polyacrylamide gel electrophoresis, followed by isolation and transfer onto polyvinylidene fluoride membranes (PVDF, Millipore, USA). After blocking, the membranes were incubated with primary antibodies overnight at 4 °C. After washing with tris-buffered saline-Tween 20, the membranes were incubated with a secondary antibody for 1 h. Enhanced chemiluminescence (BioVision) was used for protein detection, and the reactive bands were visualized using ChemStudio Imaging (Analytikjena, German). The intensities of specific bands were quantified using ImageJ software, and β-actin was used as a loading control. The primary antibodies used: anti-interleukin [IL]-1β (1:1000, Abcam), anti-IL-10 (1:1000, Abcam), anti-TNF-α (1:1000, Santa Cruz), and anti-β-actin (1:5000, Abcam).

### Statistical Analysis

All data are presented as the mean ± standard deviation. SPSS 20.0 software (version 20.0) was used for data analysis. The statistical graphs were processed using GraphPad Prism 9 (Graph Pad Software, San Diego, USA) and Adobe Photoshop CS4 software (Adobe Systems Incorporated, San Jose, CA, USA). A one-way analysis of variance was used to analyze the differences among groups and the least-significant difference test was performed for multiple comparisons. *P*<0.05 was considered statistically significant.

## Results

### Early Administration of TAK-242 Inhibited TLR4 Expression in the Hippocampus After HIBD

**Figure 1:**
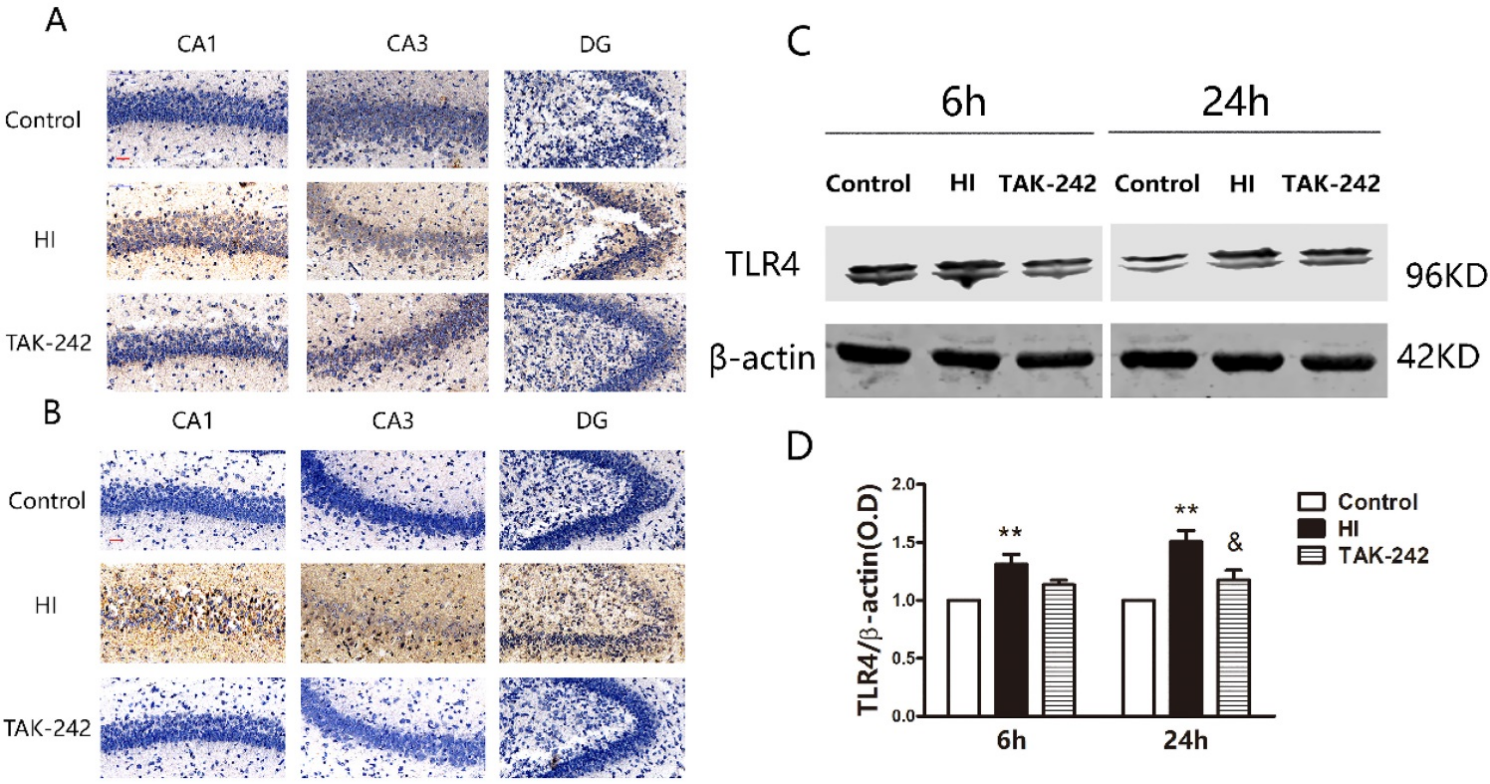
TAK-242 can decelerate the activation of TLR4 in the hippocampi of neonatal rats after HIBD. (A) TLR4 expression in the hippocampal CA1, CA3, and dentate gyrus (DG) regions at 6 h after HI. Scale bar: 20 μm, n=2-3. (B) TLR4 expression in the hippocampal CA1, CA3, and DG regions at 24 h after HI. Scale bar: 20 μm, n=3-4. (C) Western blot analysis demonstrates the differences in TLR4 expression levels in the hippocampi among groups at 6 and 24 h after HI. n=4. (D) Densitometry of the TLR4 bands is correlated with the β-actin band at 6 h and 24 h after HI. **P<0.01 vs the control group; & *P*<0.05 vs the HI group.

Immunohistochemistry showed that TLR4 was mainly expressed in cell membranes. Additionally, TLR4 expression in CA1, CA3, and dentate gyrus (DG) was higher in the HI group than in the control group, and lower in the TAK-242 group than in the HI group at 6 and 24 h after HIBD (Figure 1A, 1B).

Western blotting showed that TLR4 expression was significantly increased at 6 h and 24 h after HIBD in the ipsilateral hippocampus (*P*<0.05). TAK-242 reduced TLR4 expression at 24 h after HIBD (*P*<0.05), but no significant difference was observed at 6 h (*P*>0.05) (Figure 1C, 1D).

### Early Inhibition of TLR4 Counteracted the Increase in Iba-1+ Microglia and GFAP+ Astrocytes in the Hippocampal CA1 Region of Neonatal Rats After HIBD

**Figure 2:**
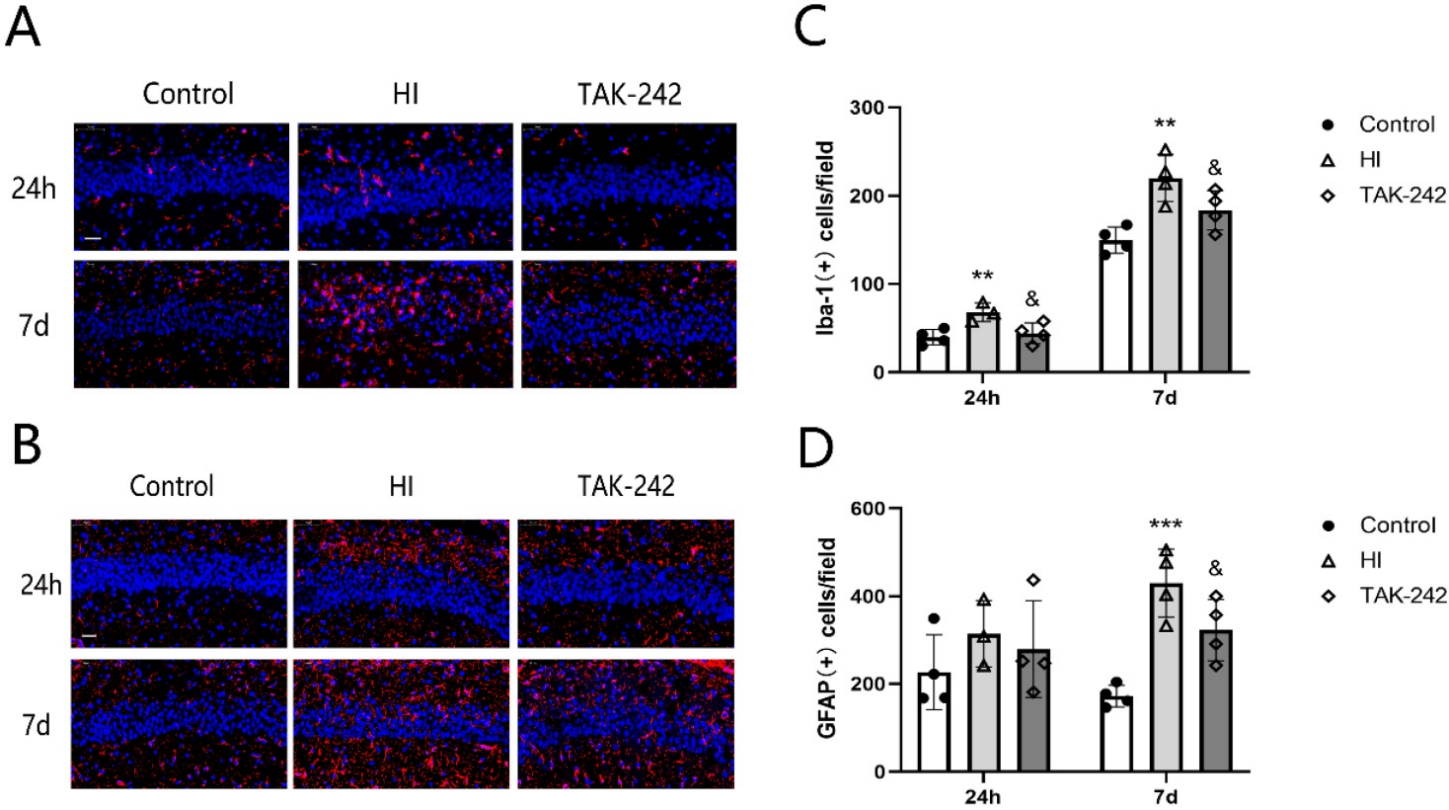
TAK-242 treatment affects the distribution of glial cells after HIBD in neonates. (A) The positive expression of Iba-1 represents microglial distribution in the hippocampal CA1 region at 24 h and 7 days after HI. Scale bar: 20 μm. (B) Positive expression of GFAP represents the distribution of astrocytes in the hippocampal CA1 region at 24 h and 7 days after HI. Scale bar: 20 μm. (C) The difference in the number of Iba-1+ cells in the CA1 region in each group. n=4. (D) The difference in the number of GFAP+ cells in the CA1 region in each group. n=4. **P<0.01, and ***P<0.001 vs the control group; &*P*<0.05 vs the HI group.

The number of Iba-1+ cells in the left hippocampal CA1 region of the HI group increased at 24 h and 7 days after HIBD (*P*<0.05), and TAK-242 counteracted this increase (*P*<0.05) (Figure 2A, 2C). The number of GFAP+ cells increased at 24 h after HIBD, and TAK-242 also counteracted this increase; however, there was no significant difference among the three groups at 24 h after HIBD (*F*=0.792, *P*=0.486). At 7 days after HIBD, the number of GFAP+ cells was higher in the HI group than in the control group (*P*<0.05), and lower in the TAK-242 group than in the HI group (*P*<0.05) (Figure 2B, 2D).

### Early Inhibition of TLR4 Counteracted the Increase MPO+ Neutrophils, CD161+ Natural Killer Cells, and CD3+ T Cells in the Hippocampal CA1 Region of Newborn Rats After HIBD

**Figure 3:**
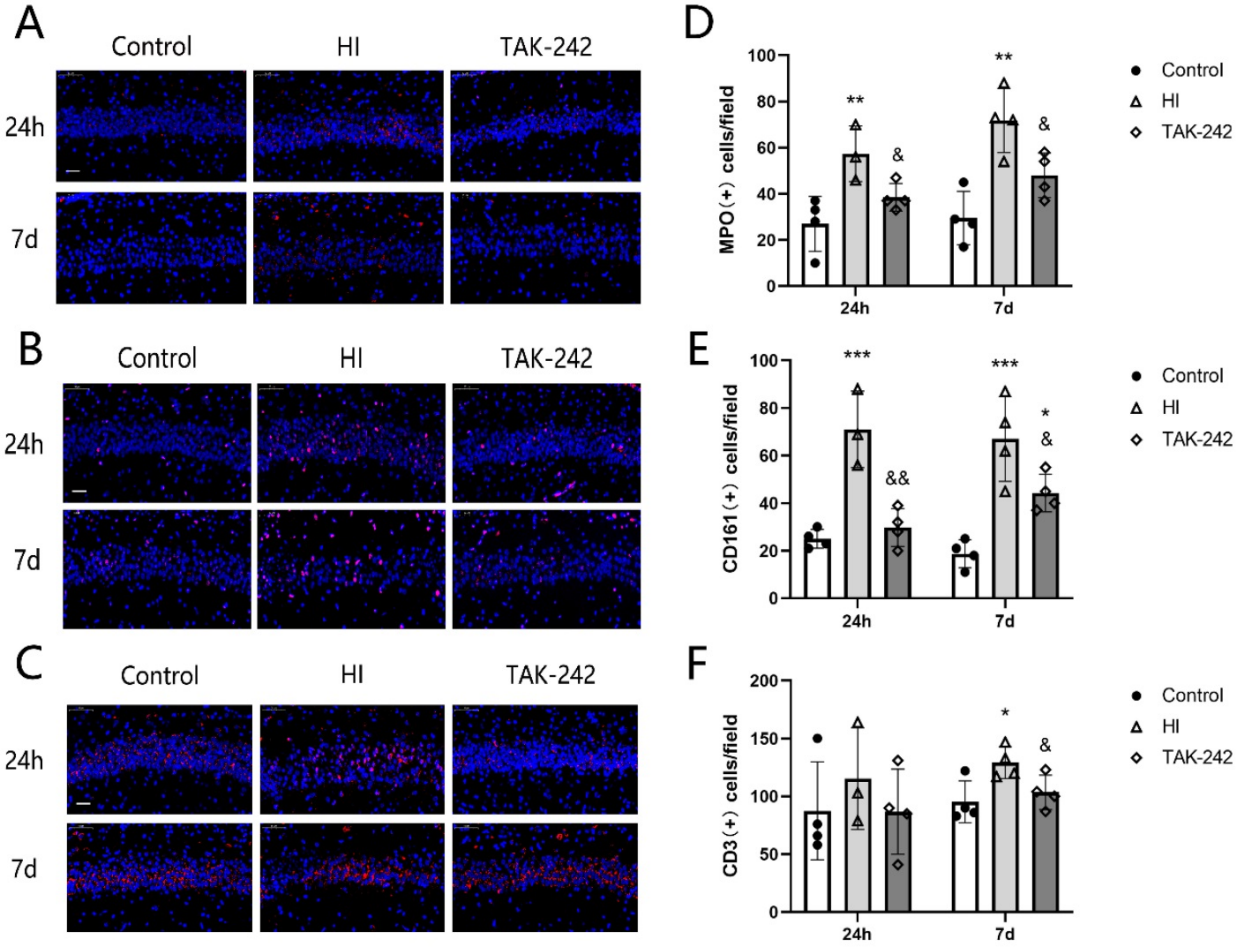
TAK-242 treatment affects the distribution of neutrophils, natural killer (NK) cells, and T cells after HIBD in neonates. (A) The positive expression of MPO represents the distribution of neutrophils in the hippocampal CA1 region at 24 h and 7 days after HI. Scale bar: 20 μm. (B) The positive expression of CD161 represents the distribution of NK cells in the hippocampal CA1 region at 24 h and 7 days after HI. Scale bar: 20 μm. (C) The positive expression of CD3 represents the distribution of T cells in the hippocampal CA1 region at 24 h and 7 days after HI. Scale bar: 20 μm. (D) The difference in the number of MPO+ cells in the CA1 region among groups. n=4. (E) The difference in the number of CD161+ cells in the CA1 region among groups. n=4. (F) The difference in the number of CD3+ cells in the CA1 region among groups. n=4. *P<0.05, **P<0.01, and ***P<0.001 vs the control group; & *P*<0.05 and &&*P*<0.01 vs the HI group.

The numbers of MPO+ neutrophils and CD161+ natural killer (NK) cells in the left hippocampal CA1 region were increased compared with the control group at 4 h and 7 days after HIBD (*P* <0.05), and TAK-242 counteracted these increases (*P*<0.05) (Figure 3A, 3B, 3D, 3E). The number of CD3+ T cells was also increased in the HI group compared to the control group at 7 days after HIBD, and TAK-242 counteracted this increase (*P*<0.05), but no significant difference was noted among groups at 24 h (*F*=0.523, *P*=0.611) (Figure 3C, 3F).

### Early Inhibition of TLR4 Counteracted the Increase in ICAM-1 Expression in the Hippocampal CA1 Region of Neonatal Rats After HIBD

**Figure 4:**
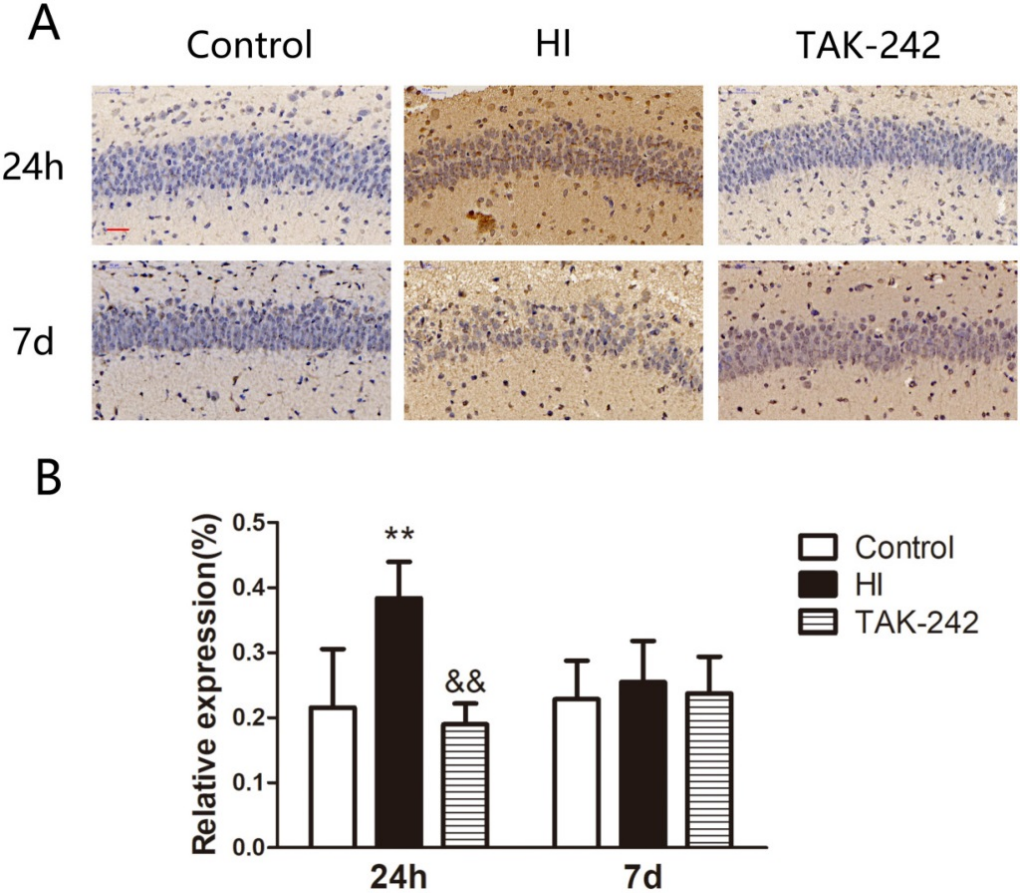
TAK-242 treatment affects the expression of adhesion factor after HIBD in neonates. (A) The positive expression of ICAM-1 represents the expressions of adhesion factor in the hippocampal CA1 region at 24 h and 7 days after HI. Scale bar: 20 μm. (B) The difference in the expression levels of ICAM-1 in the CA1 region among groups. n=4. **P<0.01 vs the control group; &&*P*<0.01 vs the HI group.

ICAM-1 expression in the left hippocampal CA1 region increased at 24 h after HIBD, and TAK-242 counteracted this increase (*P*<0.05). At 7 days after HIBD, ICAM-1 expression was higher in the HI group than in the control group, and lower in the TAK-242 group than in the HI group, although the differences were not significant (*F*=0.197, *P*=0.824) (Figure 4).

### Early Inhibition of TLR4 Counteracted the Decrease in C3a Expression in the Hippocampal CA1 Region of Neonatal Rats After HIBD

**Figure 5:**
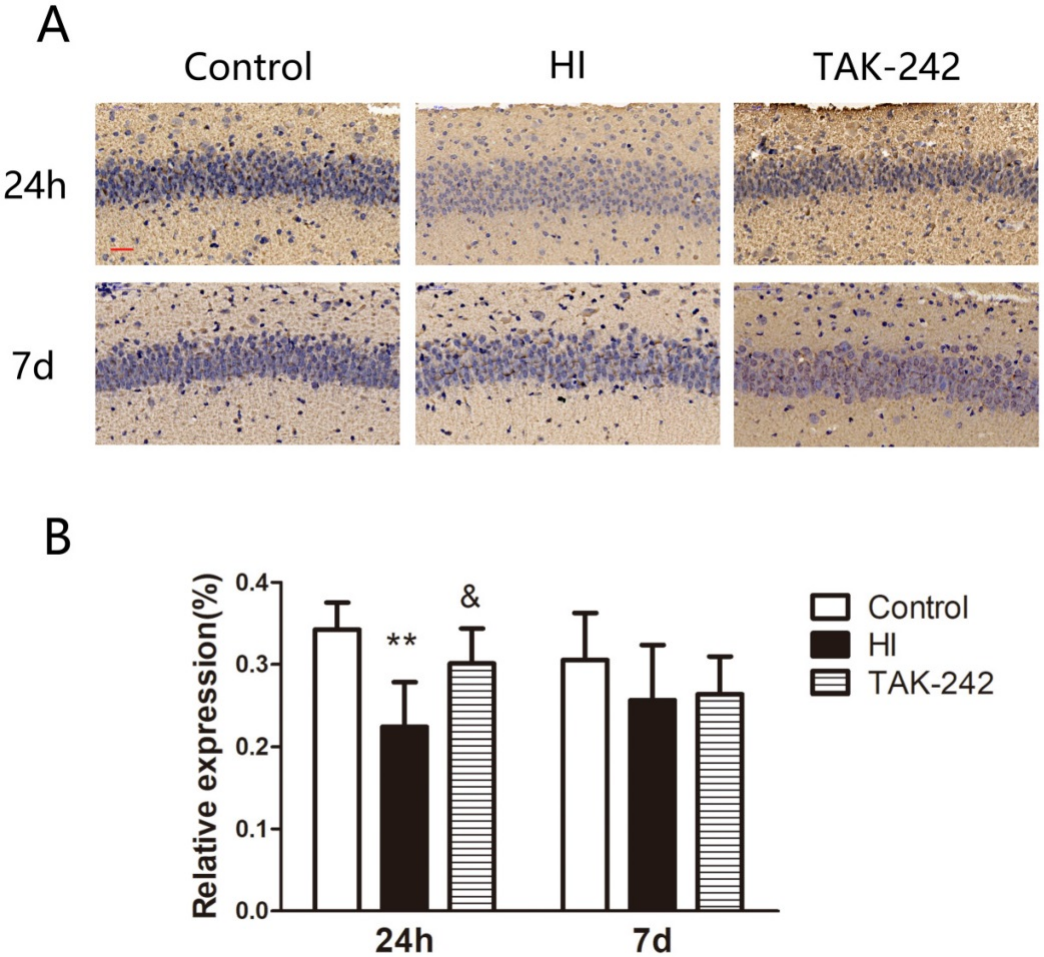
TAK-242 treatment affects the expression of complement after HIBD in neonates. (A) The positive expression of C3a represents the expression of complement in the hippocampal CA1 region at 24 h and 7 days after HI. Scale bar: 20 μm. (B) The difference in the expression levels of C3a in the CA1 region among groups. n=4. **P<0.01 vs the control group; &*P*<0.05 vs the HI group.

C3a expression in the left hippocampal CA1 region was decreased at 24 h after HIBD, and TAK-242 alleviated this decrease (*P*<0.05). At 7 days after HI, C3a expression was lower in the HI group than in the control group, and higher in the TAK-242 group than that in the HI group, but the differences were not significant (*F*=0.849, *P*=0.459) (Figure 5).

### Early Inhibition of TLR4 Affected TNF-α, IL-1β, and IL-10 Expression in the Hippocampus of Neonatal Rats After HIBD

**Figure 6:**
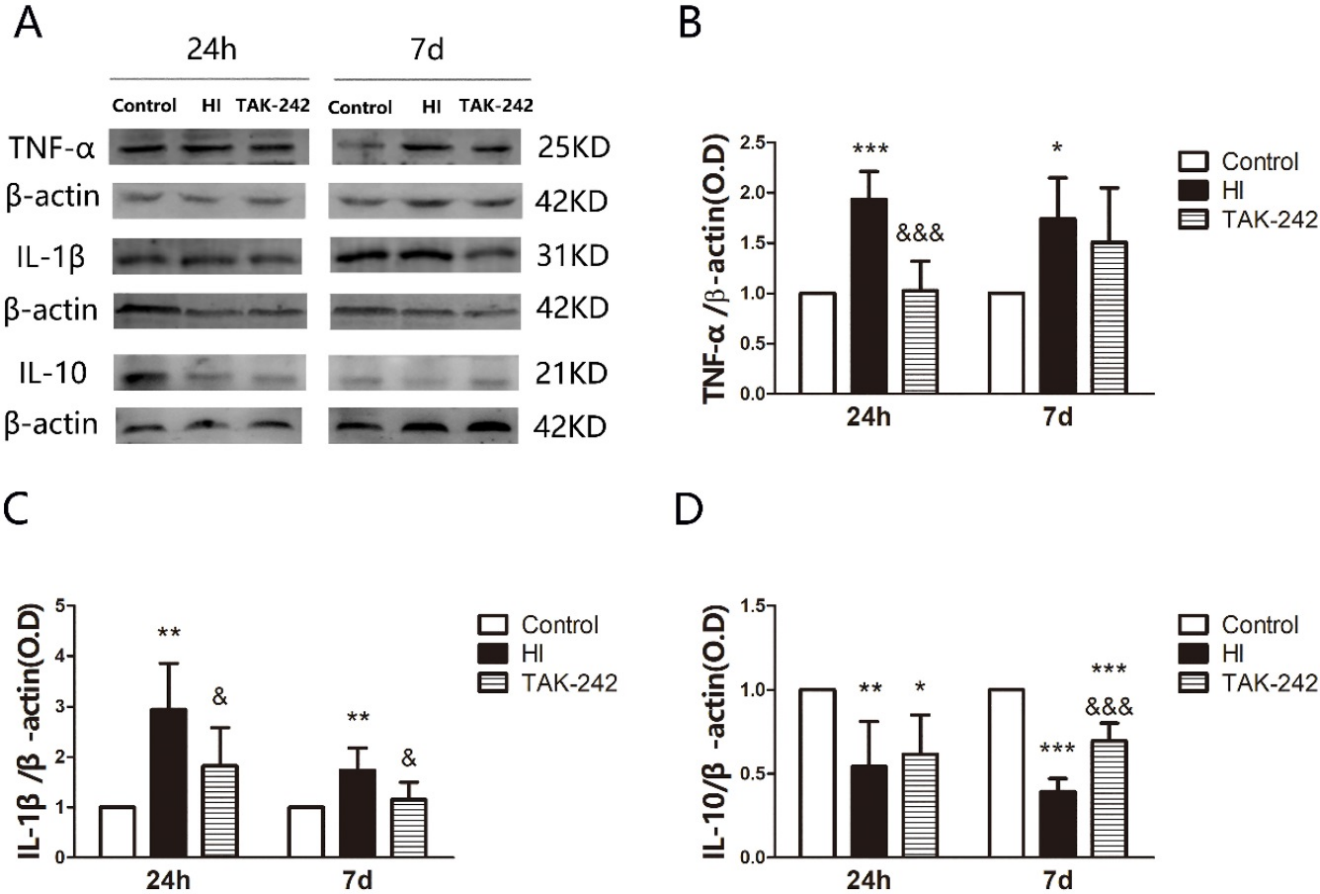
TAK-242 treatment affects the expression of inflammatory factor after HIBD in neonates. (A) The protein levels of tumor necrosis factor TNF-α, interleukin IL-1β, and IL-10 expression in the hippocampus at 24 h and 7 days after HI as determined by western blotting. Quantification of the expression levels of TNF-α (B), IL-1β (C), and IL-10 (D) in the hippocampus at 24 h and 7 days after HI. *P<0.05, **P<0.01, and ***P<0.001 vs the control group; &*P*<0.05 and &&&*P*<0.001 vs the HI group.

TNF-α expression in the left hippocampus was increased at 24 h and 7 days after HI (*P*<0.05), and TAK-242 counteracted the increase at 24 h (*P*<0.05), but there was no significant difference between the TAK-242 group and the HI group at 7 days (*P*>0.05) (Figure 6A, 6B). IL-1β expression in the left hippocampus was increased at 24 h and 7 days, and TAK-242 alleviated these increases (*P*<0.05, respectively) (Figure 6A, 6C). IL-10 expression was decreased at 24 h and 7 days (*P*<0.05). TAK-242 alleviated the decrease at 7 days (*P*<0.05), but no significant difference was noted between the TAK-242 group and the HI group at 24 h (*P*>0.05) (Figure 6A, 6D).

## Discussion

Neonatal brain development is the continuation of fetal brain development outside the uterus, immune mechanism is involved in various stages of brain development. A variety of factors including intracranial hemorrhage, infection, and hypoxia can mediate various forms of nerve cells death by inducing abnormal immune responses in the CNS, and eventually lead to acute and delayed brain damage 18. Early correction of the neuroimmune dysfunction is important for improving the prognosis after brain damage. Our main findings were as follows: (1) the early administration of TAK-242 can downregulate TLR4 expression in the hippocampi of neonatal rats after HIBD; (2) HI provokes an inflammatory cascade leading to brain damage by activating the resident immune cells, such as microglia and astrocytes; recruiting a large number of peripheral immune cells, such as leukocytes and lymphocytes; secreting a large number of adhesion factors; activating the complement system; and causing an inappropriate release of inflammatory factors; and (3) early TLR4 inhibition may improve HIBD prognosis by alleviating the HI-induced neuroimmune dysfunction, including the activation of immune cells, complement system impairment, and inappropriate expression of adhesion factors and pro-/anti-inflammatory factors.

As the main resident immune cells in the CNS, microglia play an important role in the neuroimmune response in the brain. We found that the number of microglia in the hippocampal CA1 region of neonatal rats increased significantly at 24 h after HI, which was consistent with the outcomes observed in a P7 Wistar rat HIBD model 19. Microglia in the cerebral cortex were continuously activated, accompanied by an increase in inflammatory cytokines and oxidative stress markers until 1 year after brain injury, which was related to lesion expansion, hippocampal neurodegeneration, and myelin loss 20. The continuous activation of microglia can also be observed in neurodegenerative diseases such as Alzheimer's disease and Parkinson's disease 21. In this study, we also found that the number of microglia and expression of IL-1β and TNF-α increased until 7 days after HI. Microglia recognize damage-associated molecular patterns and pathogen-associated molecular patterns via various receptors on the cell membrane to activate the immune response 22-24. Inhibiting receptors such as TLRs can reduce the brain damage caused by ischemia 14, 25. In this study, we used TAK-242 to inhibit TLR4 expression, which reduced the activation of microglia in the CA1 region of the hippocampus at 24 h and 7 days after HIBD. The upregulation of GFAP expression is an important sign of astrocyte proliferation and formation of reactive astrocytes 26. Similar to the long-term reactive astrocyte proliferation after HIBD observed in our previous study 16, we observed an increase in the number of astrocytes at 24 h after HIBD, which continued until 7 days after HIBD. TLR4 is also localized on the surface of the astrocytic membrane and plays an important role in their activation. In a lipopolysaccharide-induced P14 mouse epilepsy model, TLR4 localized on the surface of astrocytes was activated and mediated persistent epilepsy in mice by affecting synaptic plasticity; thus, TLR4 inhibition can reduce astrocyte activation and relieve brain damage 27, 28. Our study also confirmed that TLR4 inhibition can reduce astrocyte activation in the hippocampus after HIBD, implicating TLR4 as a therapeutic target for neonatal HIBD.

HI not only induces resident CNS immune cells activation but also activates peripheral immune cells, such as neutrophils, lymphocytes, and mast cells. Moreover, the activated peripheral immune cells can migrate into the CNS via the damaged blood-brain barrier and lymphatic vessels, and mediate brain damage together with resident immune cells 29. Further, neutrophils are involved in adult post-stroke brain damage and behavioral disorders 30. Previous studies suggested that neutrophils play a minor role in neonatal HIBD due to their immature function, weak response to injury, and slow migration to the injury site 31, 32. However, recent studies have shown that neutrophils can also rapidly accumulate at the brain injury site after neonatal HIBD and exacerbate HI-mediated brain damage 1, 33-35. We also found that the number of neutrophils in the hippocampus was increased at 24 h after HIBD and continued to increase for 7 days. In contrast, T-lymphocytes were observed at the brain injury site at 3 days after neonatal HI, and persisted for an extended period 36-38. Moreover, CD3+ T-lymphocytes were significantly increased in the hippocampus at 7 days after HI, suggesting that T-lymphocytes are more likely to participate in the delayed immune response. In an adult animal model of cerebral ischemia, NK cells were observed in the brain's injured tissue after HIBD and were involved in the neuroimmune response independent of T-lymphocytes 39, 40. Although the function of neonatal peripheral NK cells is immature and the neurotoxicity is weak, reducing the number of peripheral NK cells by early splenectomy demonstrated neuroprotective effects against HIBD 41. We found that the number of NK cells in the hippocampus increased significantly at 24 h after HIBD and continued to increase for 7 days, indicating that NK cells participate in the immune response. TLR4 is involved in immune cell activation and mediates brain injury by activating neutrophils, T-lymphocytes, and NK cells in the pathogenesis of stroke and sepsis 42. We found that early TLR4 inhibition could suppress neutrophil, T-lymphocyte, and NK cell activation in the hippocampus after HIBD, although the underlying mechanisms remain unclear.

Inflammatory factors, adhesion factors, and the complement system play an important role in the pathogenesis of neonatal HIBD. After brain damage, activated glial cells, endothelial cells, and peripheral immune cells can produce inflammatory factors 1, 43, 44. TNF-a and IL-1β are two important pro-inflammatory cytokines, and their increased expression is positively correlated with brain damage severity 1, 5, 19, 45-47. While secreting pro-inflammatory factors, the activated immune cells also secrete anti-inflammatory factors 1, 5. We found that TNF-a and IL-1β expression in the hippocampus increased significantly at 24 h and continued for 7 days, while IL-10 expression decreased till 7 days after HIBD.

After cerebral ischemia or hemorrhage, endothelial cells are activated and express adhesion molecules to form an adhesion surface. Peripheral immune cells roll on it, decelerate, and finally stop forming clusters at the site of brain injury 30, 48, 49. We found that ICAM-1 expression in the hippocampus increased at 24 h after HIBD. C3 is the highest complementary component in the serum and plays an important role in both the classical and alternative activation pathways of complement 50. HIBD was more severe in C3a gene-deficient mice, and early exogenous C3a treatment reduced HIBD damage 51. We also found that C3a expression in the hippocampal CA1 region decreased at 24 h after HIBD, suggesting that the complement system is involved in the process of acute brain jury after HI. TLR4 is an important pattern recognition receptor in the regulation of transcription of various inflammatory factors by activating the nuclear factor-kappa B signaling pathway. We confirmed that early TLR4 inhibition can improve the expression disorder of adhesion factors, the complement system, and pro-/anti-inflammatory factors, suggesting that TLR4 is directly involved in neuronal functional impairment after HIBD.

## Conclusion

We demonstrated the changes of immune cells and factors at 24 h and 7 d after neonatal HI, including CNS resident immune cells and peripheral immune cells, adhesion factors and pro-/anti-inflammatory factors. Our study revealed that TLR4 can mediate neuroimmune dysfunction in the hippocampus after HIBD and provided a theoretical basis for TLR4-targeted intervention for HIBD. However, the underlying mechanism has not been clarified, and the long-term immune response after HIBD was not observed; this should be further studied.

## Figures and Tables

**Table 1:** Animal categorization.

Group	Number	Male/female	Deaths	Sample size
Control	25	12/11	0	IHC & IF (n_6 h_=3, n_24 h_=4, n_7 d_=4)
				Wb (n_6 h_=4, n_24 h_=5, n_7 d_=5)
HI	25	10/15	3	IHC & IF (n_6 h_=2, n_24 h_=3, n_7 d_=4)
				Wb (n_6 h_=4, n_24 h_=5, n_7 d_=4)
TAK-242	25	15/10	2	IHC & IF (n_6 h_=3, n_24 h_=4, n_7 d_=4)
				Wb (n_6 h_=4, n_24 h_=4, n_7 d_=4)
